# Cross-domain microbiomes: the interaction of gut, lung and environmental microbiota in asthma pathogenesis

**DOI:** 10.3389/fnut.2024.1346923

**Published:** 2024-06-21

**Authors:** Jiale Zhang, Xianhui Zheng, Wenting Luo, Baoqing Sun

**Affiliations:** ^1^Department of Clinical Laboratory, National Center for Respiratory Medicine, National Clinical Research Center for Respiratory Disease, State Key Laboratory of Respiratory Disease, Guangzhou Institute of Respiratory Health, The First Affiliated Hospital of Guangzhou Medical University, Guangzhou, China; ^2^Guangzhou Laboratory, Guangzhou, China

**Keywords:** gut-lung axis, respiratory microbiome, gut microbiome, asthma, short-chain fatty acids (SCFA)

## Abstract

Recent experimental and epidemiological studies underscore the vital interaction between the intestinal microbiota and the lungs, an interplay known as the “gut-lung axis”. The significance of this axis has been further illuminated following the identification of intestinal microbial metabolites, such as short-chain fatty acids (SCFA), as key mediators in setting the tone of the immune system. Through the gut-lung axis, the gut microbiota and its metabolites, or allergens, are directly or indirectly involved in the immunomodulation of pulmonary diseases, thereby increasing susceptibility to allergic airway diseases such as asthma. Asthma is a complex outcome of the interplay between environmental factors and genetic predispositions. The concept of the gut-lung axis may offer new targets for the prevention and treatment of asthma. This review outlines the relationships between asthma and the respiratory microbiome, gut microbiome, and environmental microbiome. It also discusses the current advancements and applications of microbiomics, offering novel perspectives and strategies for the clinical management of chronic respiratory diseases like asthma.

## 1 Introduction

As microbiome research advances, it becomes increasingly clear that the gut microbiota interacts with various organs and systems in the human body, forming a complex network that collectively regulates many aspects of human metabolism. Studies have shown that disruptions in the gut microbiota are associated with a range of diseases, including respiratory disorders, metabolic conditions, and cardiovascular diseases, highlighting the microbiota's integral role in host physiology. Beyond the well-known gut microbiota, microbial communities in other regions of the body, such as the oral cavity, skin, reproductive tract, and respiratory tract, contribute to diverse functions in host regulation. These functions include pathogen inhibition, the secretion of bioactive metabolites, and immunomodulation. Furthermore, these microbiota can influence physiological processes in other parts of the body through the circulatory system (1). Although the functions of the gut and lungs differ significantly, they share a common developmental origin and possess similar structural foundations. Both are covered by a thin mucosal layer that provides lubrication and defense against pathogens. Additionally, mucosal membranes are vital components of the integrated immune network that protects the body from infection. During respiratory diseases, the strictly regulated gut-lung axis may affect various molecular patterns, potentially exacerbating the severity of the disease and leading to dysfunction in normal functions. Research has revealed significant alterations in the respiratory microbiota, including bacteria, fungi, viruses, and their critical ligands and metabolites, in patients with chronic obstructive pulmonary disease (COPD) and allergic asthma, among other chronic respiratory diseases, compared to healthy individuals. However, the precise mechanisms by which these microbial communities influence disease development remain to be fully elucidated (2). Furthermore, the gut microbiota can potentially increase an individual's susceptibility to allergic airway diseases like asthma through the gut-lung axis. Investigating the interactions between respiratory microbiota and the host, as well as their associations with different disease phenotypes, can help identify biomarkers related to disease progression and risk stratification (3). These microbial communities inhabiting various body sites may also serve as potential targets for the treatment of chronic respiratory diseases.

Asthma represents a severe global health issue, being one of the most common chronic respiratory diseases in both children and adults. It's characterized by chronic airway inflammation, involving immune cells, inflammatory cells, airway epithelial cells, and various cellular components. Key features include airway inflammation, airway remodeling, and heightened airway responsiveness (4–6). Approximately 300 million people worldwide are affected by asthma (7), with prevalence rates ranging from 1 to 18% in different countries. In China, asthma incidence is on the rise, particularly among children. Reports indicate that asthma prevalence in individuals aged 20 and older in China is 4.26%, totaling 45.7 million patients (8). Asthma results from complex interactions between environmental factors and genetic susceptibility. The composition of the human microbiome is associated with increased rates of allergic diseases (9–11). One possible mechanism involves the presence of microbial communities in fetal placenta and meconium, suggesting that contact with bacteria occurs even before birth. Early fetal exposure to microbial communities and other allergens may contribute to inducing immune tolerance to these “harmless” antigens, thus preventing allergies in children. One possible mechanism involves the presence of microbial communities in fetal placenta and meconium, suggesting that contact with bacteria occurs even before birth (12). Furthermore, postnatal breastfeeding provides infants with a rich source of secretory IgA and prebiotic oligosaccharides, which aid in microbial colonization and enhance microbial diversity (13). The long-term stability of most microbial species begins at around the age of two. Therefore, studying the composition and function of early-life microbial communities can help unravel the development and occurrence of allergic diseases, as well as environmental risk factors that might induce these conditions. Microbial communities represent a potential therapeutic target for allergic asthma, serving as both preventive measures and adjunct therapies used in conjunction with oral allergen preparations or epidermal immunotherapy (14).

The human body hosts trillions of dynamic and diverse microbial communities collectively known as the “human microbiome” (15). These communities have the potential to regulate host metabolism, maintain immune system homeostasis, and prevent pathogen invasion. They are distributed throughout various parts of the body, with the majority residing in the gastrointestinal and respiratory tracts, commonly referred to as the gut microbiome and the respiratory microbiome. Over the past decade, significant advancements have been made in the field of microbiome research, propelled by initiatives such as the Human Microbiome Project, Human Microbiome Genomic Project, and the American Gut Project. The development of metagenomic techniques has shed light on the high diversity of microbial communities within the human body, both in terms of biology and functionality. The rapid progress of high-throughput sequencing technologies has enriched the ways in which researchers explore the interactions between microbial communities and their host, better facilitating the understanding of the roles, functions, and mechanisms of these communities in the human body and even in the global ecosystem. In the future, further in-depth research into the mechanisms mediated by microbial communities holds the promise of providing new perspectives and strategies for the prevention and treatment of a range of chronic respiratory diseases, including asthma.

## 2 Respiratory microbiome and asthma

For a long time, it was widely believed that the healthy human lung was a sterile environment. However, it has now been confirmed that the lungs are indeed colonized by a unique microbial community. This phenomenon is not limited to adults; even newborns have been found to have microbial communities in their lungs (16, 17). The lung microbiome exhibits two notable characteristics. First, the microbial density in the lungs is relatively low, with the bacterial genomes hidden in the human lung estimated to be around 2.2 × 10^3^/cm^2^ (18). This density is comparable to that found in the human duodenum, which contains ~10^4^/mL microbes (19). Second, the lung microbiome is in a constant state of renewal and replacement, with the most common communities consisting mainly of the *phyla Actinobacteria, Firmicutes, Proteobacteria, and Bacteroidetes* (3, 20–22). Similar to the gastrointestinal tract, *Actinobacteria* and *Firmicutes* are considered part of the “core” microbial communities in the lungs (23). The mucosal immune system, an integral part of the human immune network, interacts with the abundant microbial communities residing on the mucosal surfaces of the respiratory tract, contributing to the maintenance of the body's homeostasis and playing a crucial role in infection defense (24). The balance of the respiratory microbiota is constituted by the dynamic process in which the microbiome migrates into the lungs through “micro-aspiration” and the host defense mechanisms clear the microorganisms. When the composition and/or function of the microbial communities on the respiratory mucosa change, the critical ligands and metabolites of bacteria, fungi, and viruses in these microbial communities can impact the regulation of the host immune system (2). This can occur by upregulating inflammatory signals (such as NF-κB, Ras, IL-17, and PI3K) or inhibiting the production of tumor necrosis factor (TNF) and IFN-γ in response to lower airway pathogens, ultimately leading to the development of chronic respiratory diseases. In mice, it has been validated that after aspiration of a mixture of oral commensals (*Prevotella melaninogenica, Veillonella parvula, and Streptococcus mitis*), lower airway immune function changes occur, leading to the activation of CD4^+^ and CD8^+^T cells and the recruitment of IL-17-producing T cells (25). However, it remains uncertain whether microbial dysbiosis is the cause of the disease or a result of the disease process. Studies have also indicated that during respiratory diseases, such as cystic fibrosis (26), chronic rhinosinusitis (27), and asthma (28), there is dysfunction of lung cilia, increased mucus secretion, and enhanced bacterial migration (e.g., gastroesophageal reflux). These conditions appear to exceed the airway's capacity to clear microorganisms, leading to increased microbial density and a disrupted balance of airway microbiota. In a prospective cohort study in Denmark conducted by Thorsen et al. (29), they analyzed the respiratory microbial community composition of 700 one-month-old children. They found that higher diversity in children's respiratory microbial communities and the high abundance of Streptococcus and Prevotella were associated with lower levels of TNF-α and IL-1β in the respiratory immune system, and elevated levels of CCL2 and CCL17. Changes in these cytokines are closely related to the development of asthma and are considered independent risk factors. This discovery reveals the relationship between the characteristics of the respiratory microbiome and the respiratory immune system, providing new evidence for the impact of early-life microbial communities and their interaction with immune factors on asthma risk.

While the precise mechanisms through which the respiratory microbiome regulates the development of diseases remain incompletely understood, extensive research has provided strong evidence linking dysbiosis in the respiratory microbiome to the onset and progression of asthma. The lungs, being a vital site that interfaces with the external environment, host a unique microbiome characteristic to lung tissues. DNA sequencing analyses have highlighted substantial differences in the microbial composition between healthy and diseased lungs (30). Compared to healthy control populations, individuals with asthma exhibit higher microbial diversity in their microbiome and altered composition, characterized by an increase in *Proteobacteria* and a decrease in *Actinobacteria*. Notably, Wang et al. (31) analyzed sputum microbiota in asthma patients, revealing a correlation between reduced abundance of Granulicatella and asthma, while confirming an increase in Streptococcus in the respiratory microbiota of asthma patients. Ariel Hernandez-Leyva et al. found that specific bacteria in the upper respiratory microbiome, including Haemophilus, Moraxella, and Streptococcus, were associated with an increased risk of wheezing and acute respiratory infections during childhood (32). Furthermore, a longitudinal multicenter cohort study conducted by Abdel-Aziz et al. analyzed the sputum microbiota from 100 severe asthma subjects, finding that individuals with a loss of core microbiota (reduced microbial richness and decreased α-diversity) exhibited more severe asthma phenotypes (33). Additionally, the composition of bronchial bacterial communities has been found to be associated with the degree of airflow obstruction and hyperresponsiveness in asthma subjects. In patients with mild asthma, bacterial diversity is negatively correlated with the degree of bronchial hyperresponsiveness (34). However, as asthma severity increases, the nature of these associations changes. It has been reported that in severe asthma, lower bacterial diversity is associated with more severe airflow obstruction, further suggesting different mechanisms of action of bacterial communities in the pathogenesis of the disease (35).

The multiple studies mentioned above unequivocally demonstrate an association between the composition and diversity of the respiratory microbiome and various asthma phenotypes (36, 37). Microbial communities can regulate immune responses in the lungs through different mechanisms, impacting the onset, phenotypic expression, severity, and treatment outcomes of asthma (5). These findings indicate that alterations in the respiratory microbiome offer potential targets for the treatment of chronic respiratory diseases. Therefore, researching specific bacteria and the structural characteristics of the respiratory microbiome contributes to the development of improved asthma diagnostics and prevention methods, offering valuable insights for translating research findings into practical applications.

## 3 Mechanisms of respiratory microbiota dysbiosis in asthma

Asthma is a complex chronic inflammatory disease that primarily affects the airways, leading to airway narrowing, difficulty breathing, and wheezing. In recent years, an increasing number of studies have shown that dysbiosis of the respiratory microbiome is closely related to the occurrence and development of asthma. Under normal circumstances, the respiratory microbiome functions to maintain immune balance and defend against pathogen invasion. However, when this microbiome becomes imbalanced, it can trigger a series of immune responses, leading to increased inflammation and airway hyperresponsiveness. Understanding the specific mechanisms of this dysbiosis is of great significance for developing new therapeutic strategies.

### 3.1 Immune system imbalance

Asthma is a chronic inflammatory disease where the immune system plays a critical role. In asthma patients, the immune response to respiratory microbiota is often abnormal, leading to excessive inflammation. Specifically, asthma is associated with an enhanced Th2-type immune response, resulting in the overproduction of IgE antibodies. These IgE antibodies bind to allergens, triggering mast cells and eosinophils to release inflammatory mediators such as histamines and leukotrienes, which further cause airway inflammation and narrowing. Moreover, the immune system in asthma patients may react inappropriately to certain microbes, complicating airway inflammation (38, 39).

### 3.2 Alterations in microbiota composition

Under normal conditions, the respiratory microbiota is diverse and balanced, maintaining a healthy airway environment. However, in asthma patients, this balance is disrupted, leading to the overgrowth of specific pathogenic bacteria. For example, *Streptococcus pneumoniae* (40) and *Haemophilus influenzae* (41) are frequently increased in the airways of asthma patients. The overgrowth of these pathogens can directly cause infections and release toxins and other harmful substances, further exacerbating airway inflammation.

### 3.3 Imbalance of metabolic products

The metabolic products of the respiratory microbiota are crucial for maintaining airway health. Normally, the microbiota produces short-chain fatty acids (SCFAs) such as acetate, propionate, and butyrate, which have anti-inflammatory properties and regulate the host immune response. In asthma patients, due to changes in microbiota composition, the production of these beneficial metabolites may decrease, leading to heightened inflammatory responses. Additionally, some pathogenic microbes may produce harmful metabolites, worsening asthma symptoms (42).

### 3.4 Compromised airway barrier function

Airway epithelial cells form the primary barrier of the respiratory tract, preventing pathogen invasion. In asthma patients, the airway epithelial barrier function is often impaired, characterized by reduced tight junction proteins and disrupted epithelial cell layer integrity. This impairment allows pathogens to enter and colonize the airways more easily, inducing and exacerbating inflammation. Dysbiosis of the microbiota further weakens the barrier function, creating a vicious cycle that makes the airways more susceptible to infections and persistent inflammation (43).

### 3.5 Host genetic factors

The genetic background of the host plays a significant role in the development and progression of asthma. Numerous studies have identified gene variations associated with asthma susceptibility. For example, variations in genes such as IL-4, IL-13, and ADAM33 (44) can affect immune responses and airway structure and function, influencing the microbiota composition and function. Genetic factors may also regulate the microbiota-host interaction by affecting the development and function of the host immune system, impacting the course of asthma.

### 3.6 Environmental influences

Environmental factors significantly impact the balance of respiratory microbiota. Allergens (e.g., pollen, dust mites), air pollutants (e.g., PM_2.5_, vehicle exhaust), and lifestyle factors (e.g., smoking, diet) can alter the composition of respiratory microbiota (45, 46). Frequent antibiotic use, in particular, disrupts normal microbiota, leading to the overgrowth of harmful bacteria. Environmental factors, combined with genetic susceptibility, can trigger or exacerbate asthma (47).

### 3.7 Microbiota-host interactions

The interaction between respiratory microbiota and host cells is a critical regulatory mechanism in asthma. Microbes can influence the host immune response by interacting with receptors on the surface of host cells. Certain probiotics, for instance, can promote anti-inflammatory signaling pathways by binding to epithelial cell receptors, protecting the host from excessive inflammatory responses. In asthma patients, however, this microbiota-host interaction may be dysregulated, resulting in abnormal immune responses and persistent inflammation (48–50).

## 4 Gut microbiome and asthma (lung-gut axis)

The gut microbiome is the most densely populated microbial community within the human body (51), primarily composed of the *Firmicutes, Bacteroidetes, Actinobacteria*, and *Proteobacteria phyla* (52). It's noteworthy that the Lactobacillus genus within the *Firmicutes phylum* is one of the most significant probiotics in the gut microbiome. Increasing evidence suggests that the *Lactobacillus* genus and its constituents can engage in bidirectional immune signaling between the gastrointestinal tract and distant organs, such as the lungs. This interaction promotes anti-inflammatory responses and ameliorates asthma-related symptoms (53–55).

While the gastrointestinal and respiratory tracts are anatomically separate, they share a common embryonic origin and structural similarities, indicating that they may interact in various ways. This bidirectional communication hub between the gut and lungs is referred to as the “gut-lung axis” (56). It emphasizes the interactions and connections between gut microbiota and lung microbiota, which can influence the immune status of both organs (50), enabling bidirectional communication and regulation, and impacting an individual's susceptibility to allergic airway diseases like asthma (57, 58). Lee-Sarwar et al. (59) analyzed the gut microbiota composition and metabolites in 110 asthmatic children, discovering that children with frequent wheezing had an enrichment of the *Veillonella* genus and metabolites related to the histidine pathway in their gut. Conversely, bacterial abundances of other groups like Spirochaetaceae UCG-005, Holdemanella, and Mollicutes were negatively correlated with wheezing. These findings offer insights into further research on the mechanisms of the gut microbiota's role in asthma. Early-life dysbiosis in the gut microbiota of newborns is associated with an increased risk of developing asthma during childhood. The potential mechanism behind this is the elevated concentration of 12,13-diHOME produced by specific gut microbiota enzymes in newborns predisposed to asthma, which may reduce the number of lung Treg cells, inhibit dendritic cell anti-inflammatory factor secretion, and lead to metabolic dysfunction, potentially increasing the risk of asthma (60). Similarly, children who develop asthma during school age were found to have lower gut microbiota diversity before the age of 1 month (61). Colonization of Veillonella (*Firmicutes phylum*) at 1 month of age was also associated with wheezing and asthma at 6–7 years of age (62). Although the direct causal relationship between gut microbiota and asthma is not yet established, these studies show that changes in early-life microbial communities and specific components can affect a child's susceptibility to asthma and may trigger specific responses later in development.

**Figure 1:**
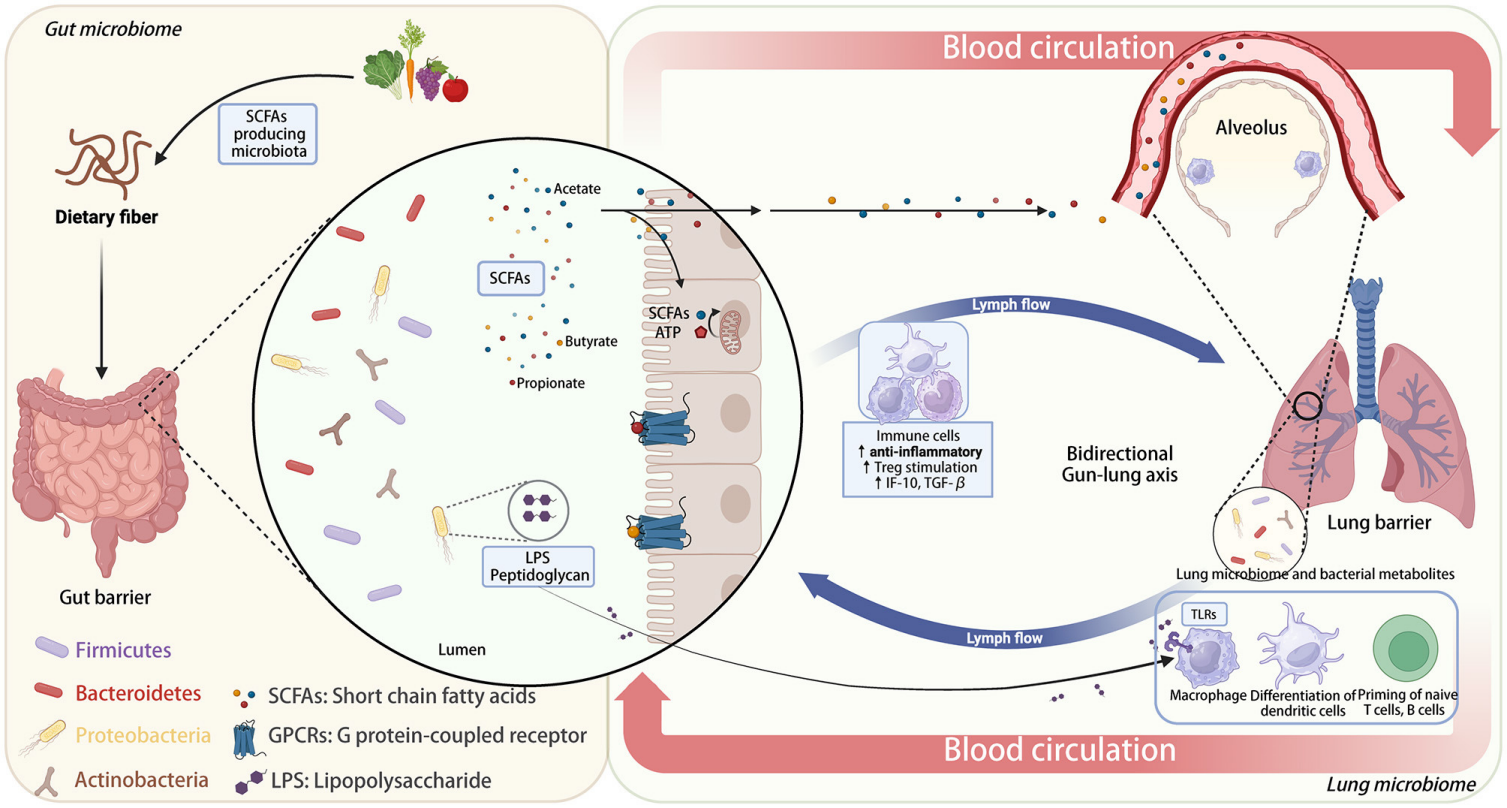
Bidirectional gut-lung axis: the role of dietary fiber and microbiota-derived SCFAs in immune modulation.

Current research has identified two primary categories of communication pathways within the gut-lung axis (63, 64): (i) Direct Impact of Gut Microbiota: This includes the presence of molecules like peptidoglycans and lipopolysaccharides (LPS), which can enhance the host's immune responses. (ii) Indirect Impact of Gut Microbiota: (a) Digestive Byproducts Entering the Circulatory System: Metabolites produced during digestion in the gut can enter the peripheral bloodstream and affect the development of immune cells. (b) Production of Short-Chain Fatty Acids (SCFA): Certain gut microbes metabolize dietary fiber to generate SCFAs, which can then trigger immune responses in the host's lungs. Unmetabolized SCFAs can also affect lung immune responses either by directly migrating to the lungs and enhancing G protein-coupled receptor (GPCR) activation (65) or by migrating to the bone marrow and promoting the differentiation of macrophages and dendritic progenitor cells into anti-inflammatory alternatively activated macrophages (AAMs). AAMs are a subtype of macrophages with immune-regulating capabilities that can reduce neutrophil recruitment and stimulate the production of anti-inflammatory cytokines (e.g., IL-10 and TGF-β) from Treg cells, thereby reducing lung damage and inflammation (54, 58). Besides, SCFAs can also modulate the metabolic pathways of alveolar macrophages exposed to LPS, a process critical for maintaining pulmonary immunometabolism (66). Furthermore, the detection of SCFAs in sputum can confirm the connection between the gut and the lungs (67, 68). A deeper understanding of the intricate mechanisms by which SCFAs, derived from gut metabolism, exert their complex effects holds promise for the development of more effective therapeutic interventions for chronic respiratory diseases (69). However, further extensive experimental and clinical validation is still needed. (c) Migration of Gut Immune Cells: Immune cells from the gut can directly migrate to the lungs via the peripheral circulatory system, influencing lung immune activities. Studies have shown that interleukin-25 (IL-25) can induce type 2 innate lymphoid cells (ILC2) to migrate from the gut to the lungs, participating in Th2-type immune responses and contributing to lung inflammation (70, 71). (d) Exchange via Lymphatics: Both gut and lung microbiota can communicate through lymphatic circulation (23, 58, 64, 71). This bidirectional communication is facilitated by chemical signals generated directly by microbiota or because of immune triggers. These signaling molecules can circulate systemically through blood and lymphatic fluids, regulating the host's immune system (72, 73). A comprehensive understanding of the complex mechanisms underlying the effects of SCFAs on the gut-lung axis can aid in the development of more effective interventions for chronic respiratory diseases. Nevertheless, further experimental and clinical validation is still required to explore these interactions (Figure 1).

## 5 Environmental microbiome and asthma (external intake)

The hygiene hypothesis posits that reduced exposure to microbes in early childhood due to increased sanitation and hygiene practices may contribute to the rise in allergic diseases, including asthma. Studies have shown that children who grow up on farms or in rural environments, where they are exposed to a diverse range of environmental microbiota, have a lower prevalence of asthma compared to children in urban settings. The exposure to various microorganisms is thought to help in the proper development of the immune system, reducing the risk of developing asthma (74–76). The microbial flora colonized in human respiratory tract and environmental microbial flora are closely related to the prevalence of allergic asthma (77, 78). One prospective cohort study involving nearly 400 children and spanning a 10-year follow-up period found that the composition and richness of indoor microbiota in homes were inversely correlated with children's asthma risk. Furthermore, there were significant differences in the indoor microbiota composition between asthmatic and non-asthmatic children. The abundance of Streptococcus was positively correlated with asthma incidence, while the abundance of Actinomycetales was negatively correlated with asthma and could largely explain the association between indoor microbiota richness and asthma (79). Farm environments have served as a model for studying determinants of early-life allergies. Epidemiological research conducted in these settings suggests that prenatal exposure may be related to the early activation of the fetal immune system. Specifically, sustained maternal exposure to microbial compounds during pregnancy appears to be associated with a reduced risk of atopic sensitization and disease outcomes in offspring (80, 81). Exposure to pets, especially during early childhood, has been associated with a reduced risk of asthma. Studies suggest that children exposed to pets such as dogs and cats have a more diverse gut and respiratory microbiota, which may help in modulating the immune response and protecting against asthma (82, 83). Similarly, exposure to livestock on farms has been shown to have a protective effect against asthma and allergies (84).

The interaction between the human gut and its microbial communities is not only regulated by environmental changes but is also closely associated with dietary habits. These two factors together lead to a wide array of bacterial selection (85). This selection may result in changes in the composition and abundance of bacterial communities, affecting the ecological functions and interactions of microbes in different environments. Research suggests that dietary factors play a significant role in the development of allergic diseases. High-fat diets that induce obesity can trigger or exacerbate asthma. In contrast, diets rich in dietary fiber can modulate lung immunity. The intake of high dietary fiber not only alters gut microbiota but also affects the microbial composition in the lungs, indicating the regulatory role of nutritional factors in maintaining lung immune homeostasis by inhibiting the activation of Th2 effector cells to alleviate allergic airway inflammation (86). Moreover, diverse gut microbiota is associated with better health outcomes. Increasing microbial diversity or the abundance of certain beneficial bacteria can reduce the occurrence of allergic symptoms (87). These findings underscore the potential benefits of diverse diets, highlighting the pivotal role of both diet and gut microbiota composition in shaping lung immune responses. Common protective dietary factors include polyunsaturated fatty acids (Omega-3, eicosapentaenoic acid, and docosahexaenoic acid), dietary fiber, SCFAs, vitamins A and D, and zinc (88–90). These components play a certain role in the prevention or alleviation of allergic asthma (91). By studying lung microbiota, the interaction between lung and gut microbiota, and their relationship with different disease phenotypes, we can identify biomarkers for disease progression and risk stratification.

In sum, other epidemiological evidence strongly supports the role of environmental microbiome exposure in the development and regulation of asthma. Factors such as urban vs. rural living, exposure to pets and livestock, antibiotic use, mode of birth, breastfeeding, air pollution, and diet all play significant roles in shaping the respiratory and gut microbiomes, thereby influencing the risk of developing asthma. Understanding these associations can help design preventive strategies and interventions to reduce the burden of asthma.

## 6 Application of microbial sequencing technology in asthma

In microbiome research, the analysis of microbial community composition primarily relies on two high-throughput approaches: amplicon sequencing (16S/18S/ITS) and whole metagenome shotgun sequencing (WMS) (92). Among these, metagenomics and meta-transcriptomics are advanced techniques using shotgun sequencing methods. They employ Microbiome-Wide Association Studies (MWAS) to reveal the taxonomic composition, functional metabolic profiles, and active expression profiles of the entire microbial community comprehensively and precisely at the DNA/RNA levels. Compared to amplicon sequencing based on genes like 16S, 18S, or ITS, metagenomics genuinely explores the ecological roles and underlying mechanisms of microbial communities in ecosystems. It delves deeper into microbial ecological questions at the functional level and represents the cutting-edge technology and essential methodology in microbiome research.

The 16S and ITS rRNA (Internal Transcribed Spacer ribosomal RNA) sequencing are common amplicon sequencing methods used to identify bacteria or fungi in a given sample. The 16S rRNA gene is prevalent in bacterial cells and is located in the ribosomal small subunit (~1,540 base pairs), which comprises nine variable regions and ten conserved regions. The conserved regions are used to design primers for targeted amplification, while analysis of the variable regions enables the identification of genera or species in different microbial communities, allowing for systematic evolutionary classification. Currently, regions used for deep sequencing of the 16S rRNA gene include V4, V3-V4, and V4-V5 (93), making it well-suited for bacterial phylogenetic studies and species identification. The ITS1 region of the rRNA subunit is a common DNA marker used to identify fungal species in metagenomic samples (94). Next-generation sequencing (NGS) of ITS and 16S rRNA genes allows for systematic phylogenetics and classification comparisons of complex microbiomes or samples that are otherwise challenging or impossible to study, making them mature techniques in this application field. However, amplicon sequencing does have its limitations, such as amplification biases, off-target amplification, and low species resolution, and it typically cannot simultaneously detect bacteria, archaea, and fungi. Shotgun metagenomic sequencing, on the other hand, can comprehensively detect all genes from every organism present in a given complex sample, allowing for the assessment of bacterial diversity and detection of microbial abundance across various environments. Moreover, it can also be employed to study unculturable microorganisms, which are challenging to analyze using other methods. NGS-based shotgun metagenomic sequencing can combine multiple samples in a single sequencing run, each achieving high sequencing coverage, enabling the detection of extremely low-abundance organisms within microbial communities. Due to the characteristic of directly fragmenting gene sequences, this technique effectively mitigates amplification bias. Moreover, theoretically, it does not limit the analysis to specific species, potentially yielding information on new genes and even new species. In conclusion, the advantages of meta-transcriptomics include moving beyond the confines of DNA, allowing for real-time insights into active genes and pathways contributing to community functions. Nevertheless, shotgun sequencing demands high DNA quality and quantity from samples, making it difficult to analyze highly degraded or severely contaminated specimens, with relatively higher sequencing costs. To overcome the core limitations of amplicon sequencing and shotgun sequencing, some studies have proposed a simplified shotgun metagenomics technology called 2bRAD-M, which can efficiently isolate microbial samples with minimal, highly degraded, or heavily contaminated from the human body and the environment. The principle behind 2bRAD-M involves processing total DNA samples from microbial communities, amplifying and sequencing cut fragments with IIB enzymes. Then, it uses the theoretical enzyme cleavage sites on various microbial genomic sequences to infer microbial community structures (95). Metatranscriptomic sequencing involves large-scale high-throughput sequencing of transcripts from all microbial communities in a specific environment at a specific time. This approach directly captures transcriptome information from both culturable and non-culturable microorganisms in the environment. Not only does this technology possess all the advantages of metagenomics, enabling the detection of active microorganisms, active transcripts, and active functions in the environment, but it also allows for the comparison of differentially expressed genes and functional pathways across different environments. This comparison reveals the adaptation mechanisms of microorganisms under various environmental pressures and explores the interaction mechanisms between the environment and microorganisms (Table 1).

**Table 1:** Common microbiome sequencing techniques.

**Technology**	**Sequencing content**	**Sequencing depth**	**Application scope**	**Advantages**	**Disadvantages**
16S rRNA Sequencing	16S rRNA gene fragments	Relatively shallow	Microbial community structure analysis, taxonomy	Low cost, simple operation, well-established databases	Limited resolution, amplification bias, off-target amplification, cannot distinguish at the species level
Metagenomics	All microbial genes	Deep sequencing (tens of Gb to several Tb)	Functional analysis of microbial communities, metabolic pathway studies	High resolution, identifies microbial species and functional genes	High cost, complex data analysis
Metatranscriptomics	mRNA from environmental samples	Moderate to deep	Gene expression and functional activity of microbial communities	Reveals dynamic functional activities of microbes	RNA stability issues, prone to degradation, complex data processing

Traditional microbial diagnostic techniques include the use of culture media (substrates and agar), serological examinations for pathogen-related antibodies, and the use of PCR technology to detect microbial genetic material (DNA or RNA). However, these techniques have significant limitations regarding their scope of application and detection sensitivity. Specifically, traditional microbial culture techniques typically require bacteria or fungi to be cultivated in media-rich environments, potentially rendering them unable to culture certain microorganisms present in extraneous environments. Furthermore, some microorganisms exist at extremely low concentrations in natural environments, making them challenging to detect using conventional culture methods. In some cases, culturing bacteria or fungi may take a significant amount of time, which is impractical for situations that require the rapid determination of the presence of pathogens, such as in infectious disease diagnostics. Hence, the next generation of RNA sequencing (RNA-Seq) for bacteria, viruses, and other microorganisms has become the standard method for analyzing transcriptome and meta-transcriptome information. RNA-Seq technology does not depend on culture or specific DNA sequences but directly detects RNA molecules within microbial samples. In addition, RNA-Seq provides critical information about microbial activity, metabolic pathways, and gene expression, which is vital for understanding microbial ecology, physiology, and pathogenicity. Furthermore, microbial transcriptome and meta-transcriptome information is crucial for predicting antibiotic resistance, understanding host-pathogen immune interactions, quantifying changes in gene expression, and tracking disease progression.

## 7 Conclusions and prospects

At present, the exact mechanisms underlying the pathogenesis of allergic asthma remain incompletely understood. Clinically, patients often experience recurrent and episodic symptoms, necessitating long-term use of symptomatic drugs such as corticosteroids, antihistamines, and bronchodilators to manage the condition. However, these symptomatic treatments do not provide long-term effectiveness and carry potential risks of drug resistance and side effects (96). Hence, there is an urgent need for in-depth research into the pathogenesis of allergic asthma to identify new therapeutic targets. Over the past decade, there has been a growing consensus that respiratory, gut, and environmental microbiota play pivotal roles in the development and progression of asthma and allergic diseases. Targeting respiratory and/or gut microbiota offers potential avenues for the prevention and management of allergic asthma and other chronic respiratory diseases (97). Notably, the integrating multi-omics analysis techniques is beneficial for uncovering the functional effects and potential mechanisms of gut microbiota in chronic respiratory diseases. This aids in exploring the intricate gut-lung axis and accelerates research into the complexity of interactions between resident microbiota and mucosal immune systems. The highly diverse microbial ecosystem within the human gastrointestinal tract exerts profound influences on the host's immune, metabolic, endocrine, and other physiological processes, all of which are interconnected. Nonetheless, the mechanisms involving host cell functional or genomic changes induced by gut microbiota and the potential involvement of gut microbiota metabolites in the pathophysiology of lung diseases remain largely unknown. The causality between gut microbiota and most lung diseases is yet to be determined. Further research in this domain is needed to unveil these complex interactions.

Therefore, by advancing our understanding of the role of microbiota in the development of asthma, it becomes possible to further evaluate the critical roles played by microbiota in human chronic airway inflammation. This has the potential to lead to the development of novel therapeutic strategies involving dietary interventions (SCFA), probiotics (*Firmicutes, Bacteroidetes, Proteobacteria, Actinobacteria*), fecal microbiota transplantation, or selective bacterial manipulation to modify microbiota (98). This review posits that investigating the relationship between microbiota changes and the development of lung diseases provides an avenue to identify new therapeutic targets. This is poised to yield significant breakthroughs in targeted screening models and microbiota-based therapies, offering fresh perspectives and strategies for clinical practice in the management of chronic respiratory diseases.

## Author contributions

JZ: Writing – original draft. XZ: Writing – original draft. WL: Writing – review & editing. BS: Writing – review & editing.
